# Concomitant laparoscopic urological procedures: Does it contribute to morbidity?

**DOI:** 10.4103/0972-9941.58500

**Published:** 2009

**Authors:** Kamlesh Maurya, S E Sivanandam, Sudhir Sukumar, Sanjay Bhat, Ginil Kumar, Balagopal Nair

**Affiliations:** Department of Urology, Amrita Institute of Medical Sciences, Kochi - 682 041, India

**Keywords:** Laparoscopy, simultaneous, urology

## Abstract

**AIM:**

With advancement in minimal access surgery two laparoscopic procedures can be combined together shortening the total hospital stay, decreasing morbidity and overall reduced cost. Combining two laparoscopic procedures in a single session has been reported in general surgery. Very few articles are available in literature with regard to combined urological laparoscopic surgeries. This article retrospectively analyses the outcomes of multiple laparoscopic procedures performed in a single stage at our centre.

**MATERIALS AND METHODS:**

Patients undergoing simultaneous procedures from May 2003 to Jan 2009 were included in the study. Patients were categorised into three groups according to the primary urological organ involved, for better comparison with the control group. Diseases involving the adrenals gland were grouped in (group 1), kidney (group 2) and renal collecting system/ureter (group 3). All patients had one urological procedure for either of the above-mentioned organs combined with another surgical procedure. Similarly three control groups were chosen according to the primary urological organ involved (group 1c- unilateral laparoscopic adrenalectomy, group 2c- unilateral laparoscopic radical nephrectomy and group 3c- unilateral laparoscopic ureterolithotomy) for comparative study. The operative details, hospital stay and complications were analysed.

**RESULTS:**

Thirty-two patients underwent 64 laparoscopic procedures under single anaesthesia. The most common procedure in this series was laparoscopic adrenalectomy (n=34) followed by laparoscopic nephrectomy (n=13). Group 1 patients had a prolonged operative time (*P* = 0.012) and hospital stay (*P* = 0.025) when compared with group 1c. However, blood loss was comparable in both the groups. Patients in groups 2 and 3 had comparable operative times, blood loss and recovery period with respect to their controls. Intraoperatively, the end tidal carbon dioxide levels were within permissible limits. All procedures were completed using the laparoscopic approach, without any conversion.

**CONCLUSIONS:**

Simultaneous laparoscopic procedures can be done for urological diseases in selected patients with the advantages of single anaesthesia and hospital admission without increasing the morbidity.

## INTRODUCTION

With advancement in laparoscopic surgery, combined laparoscopic procedures are now being performed for treating coexisting abdominal pathologies simultaneously. Simultaneous laparoscopic procedures for general surgical diseases have received good acceptance in world literature.[1–5] There have also been sporadic reports[6–8] of multiple laparoscopic procedures being combined for urological conditions and few short series[9,10] demonstrating the feasibility of dual laparoscopic procedures in urology. The advantages of simultaneous intervention are: Reduced psychological stress, single anaesthesia, less medication, shorter hospital stay and considerable cost effectiveness. Prolonged operative time with attendant haemodynamic alterations due to the pneumoperitoneum was considered to be a limiting factor for the performance of concomitant procedures. Nevertheless, once the learning curve and the necessary expertise are achieved this problem can be overcome easily. The aim of this study is to analyse the feasibility of combining multiple laparoscopic procedures during elective urologic surgery.

## MATERIALS AND METHODS

We retrospectively reviewed all concomitant laparoscopic urologic procedures done at our centre during the period between May 2003 and January 2009. The inclusion criteria for the study group were (1) both the procedures should have been done by laparoscopic approach under a single anaesthesia and (2) at least one of the procedures should have been for a urological disease.

Combined laparoscopic operations in our series were done for diseases involving the upper urinary tract (kidney and renal collecting system) and the adrenal gland. These patients were broadly divided into three groups for better comparison with the control group: Group 1 consisted of patients who underwent laparoscopic adrenalectomy combined with another laparoscopic procedure. Group 2 had patients who had undergone laparoscopic nephrectomy combined with another surgical procedure. Laparoscopic procedures involving the renal pelvis/ureter that were combined with another procedure were included in group 3. Similarly, the control group was divided into three groups according to the urological organ involved. The parameters in each group were then compared with the control groups who had undergone unilateral laparoscopic adrenalectomy, consisting of 35 patients (group 1c), unilateral laparoscopic nephrectomy with 51 patients (group 2c) and unilateral laparoscopic ureterolithotomy consisting of 18 patients (group 3c), respectively.

The computerised database of these patients was analysed with respect to the operative time, blood loss, hospital stay, complications and cost analysis. The student *t* test was used for statistical analysis of the continuous variables. The operative duration was defined as the time taken from the placement of the first port to the closure of the last port and this also included the time spent in changing the position of the patient for bilateral procedures.

The various indications for simultaneous procedures are listed in Table 1.

**Table 1:** Indications for concomitant laparoscopic procedures

Groups	Indications	No. of patients
1 (n = 18)	Cushings disease†	4
	Cushings syndrome*	9
	Bilateral phaeochromocytoma	3
	Adrenal tumour with calculus cholecystitis	1
	Adrenal tumour with simple renal cyst	1
2 (n = 9)	End-stage renal disease with uncontrolled hypertension	4
	Nephrectomy with calculus cholecystitis	3
	Non-functioning kidney with undescended testis	1
	Non-functioning lower moiety kidney with ovarian cyst	1
3 (n = 5)	Upper ureteric calculus with simple renal cyst	2
	Renal pelvic calculus with simple renal cyst	1
	Bilateral lower ureteric calculi with obstruction	1
	Pelviureteric junction obstruction with inguinal hernia	1
	Total	32

† After hypophysectomy/radiotherapy for pituitary tumour

* Ectopic ACTH production and Bilateral adrenal hyperplasia

In group 1, patients with suspected Cushing's syndrome and phaeochromocytoma underwent complete preoperative evaluation, with urine and serum biochemistry. Contrast enhanced computed tomography scan (CECT) or Magnetic resonance imaging was used for imaging the adrenal gland. Preoperatively, all patients with suspected phaeochromocytoma were hydrated well after insertion of a central line, and adequate alpha and beta blockers were given to control the blood pressure. Patients in group 2 had been evaluated with CECT abdomen to establish a diagnosis of renal mass and also to assess the local extent of the tumour. In group 3, patients with urolithiasis were evaluated with renal function tests, urine cultures, X-rays, ultrasound abdomen and intravenous urograms.

Coexisting medical morbid conditions, such as, coronary heart disease, severe obstructive airway disease and severe renal failure were considered as relative contraindications to perform a combined laparoscopic procedure.

Any variations in the port placement and extra ports, if needed, were made according to the surgical procedure. Patient position was changed intraoperatively for contralateral procedures. For patients in whom the expected operative duration was long, the end tidal carbon dioxide levels (ETCO_2_) were serially monitored during the procedure.

The advices and services of the other specialists, such as, the surgical gastroenterologist, endocrinologist and critical care anaesthesiologist were requested as and when required.

## RESULTS

Of a total of 534 patients who underwent laparoscopic procedures during the five years, 32 patients underwent 64 concomitant laparoscopic surgeries. Table 2 shows the details of simultaneously performed procedures in the study groups as well as control groups.

**Table 2:** Details of laparoscopic procedures performed in the study and control groups

Groups	Laparoscopic Procedures	No of patients
1 (n = 18)	Bilateral adrenalectomy	16
	Adrenalectomy + deroofing of renal cyst	1
	Adrenalectomy + cholecystectomy	1
2 (n = 9)	Bilateral pretransplant nephrectomy	4
	Radical nephrectomy + cholecystectomy	3
	Simple nephrectomy + orchidectomy	1
	Heminephrectomy + oophorectomy	1
3 (n = 5)	Ureterolithotomy + deroofing of renal cyst	2
	Pyelolithotomy + deroofing of renal cyst	1
	Bilateral ureterolithotomy	1
	Pyeloplasty + inguinal hernia repair	1
	Total	32
1c	Unilateral adrenalectomy	35
2c	Unilateral radical nephrectomy	51
3c	Unilateral ureterolithotomy	18

The most common operation performed was laparoscopic adrenalectomy (n = 34) done in 18 patients. Sixteen patients underwent bilateral simultaneous laparoscopic adrenalectomy and the other two had unilateral adrenalectomy combined with other laparoscopic procedures. The most common indication for laparoscopic adrenalectomy in group 1 was Cushing's syndrome (including Cushing's disease). Three of the patients underwent total bilateral laparoscopic adrenalectomy for bilateral pheochromocytoma and two of them were part of the multiple Endocrine Neoplasia Syndrome.

Laparoscopic nephrectomy including simple, radical or heminephrectomy (n = 13) was the second most common procedure. Four patients in group 2 underwent simultaneous bilateral pretransplant nephrectomy for uncontrolled hypertension, which was followed four weeks later by allograft renal transplantation. In group 3, four of the patients underwent laparoscopic procedures for urolithiasis, while one underwent a laparoscopic dismembered pyeloplasty. Of the 64 concomitant laparoscopic procedures analysed, only five were for non-urologic pathology (three - cholecystectomies, one - oophorectomy and one - hernia repair).

A majority of the procedures were performed using the transperitoneal approach; the retroperitoneal approach was used in only eight procedures. These were all right-sided and included four pre-transplant nephrectomies, two adrenalectomies, deroofing of a renal cyst and a ureterolithotomy.

The mean operative duration, mean blood loss, mean duration of hospital stay and the mean expenditure in the three groups and their controls are shown in Table 3.

**Table 3:** Comparison of intra- and postoperative data in all groups

Parameters	Group 1	Group 1c	*P* value	Group 2	Group 2c	*P* value	Group 3	Group 3c	*P* value
Mean operative time (minutes)	208	118	0.012*	188	189	0.512	165	117	0.088
Mean Blood loss (ml)	71	64.5	0.767	107	159	0.313	65	66.5	0.731
Mean hospital stay (days)	6.5	3.6	0.025*	4.5	5.0	0.616	5	4.5	0.608
Mean expenditure (thousand rupees)	38	35	-	35	31	-	33.5	28	-

* Significant *P* value

The operative duration in group 1 was almost twice that in group 1c (*P* = 0.012), which was statistically significant. However, patients in groups 2 and 3 had comparable operative durations with respect to their control arms. Also in our series, the mean estimated blood loss was comparable to that of the respective control arms in each group. The mean postoperative hospital stay was significantly prolonged in those who underwent concomitant procedures with laparoscopic adrenalectomy (*P* = 0.025), while it was comparable in the other two groups.

The mean value of ETCO_2_ for combined procedures was 36 mm Hg. Only one female patient with Cushing's syndrome had a high level of ETCO_2_ and she required overnight ventilation. Oral intake was started in the postoperative period according to the surgical procedure and the associated morbidity of the patient. In most cases, oral liquid intake was resumed within four hours of the termination of surgery and normal diet was started on the first postoperative day.

Three patients had postoperative pyrexia due to atelectasis, which improved with chest physiotherapy, steam inhalation and appropriate antibiotics. One patient with bilateral adrenalectomy for Cushing's syndrome developed reactive pleural effusion, which subsided without aspiration. Another patient had developed chylous leakage a week after bilateral laparoscopic adrenalectomy for bilateral phaeochromocytomas. There was no conversion to open and no mortality in our series.

## DISCUSSION

Laparoscopy has emerged as an advanced tool for the newer generation of urologists to treat various urological diseases. Early postoperative recovery, early return to work and better cosmesis make laparoscopy an attractive option for patients as well as for surgeons. These benefits have now been extended for the performance of combined procedures either for two different diseases or for a bilateral presentation of the same disease. More expertise, skills and hands-on training are required to accomplish the task.

If two procedures are performed simultaneously, not only do patients have the advantage of minimal access surgery, but they also have a single hospital admission, preoperative evaluation and anaesthesia exposure.

Molmenti *et al.*,[11] reported five cases of laparoscopic donor nephrectomy combined with other surgical procedures such as adrenalectomy, cholecystectomy, colposuspension and liver biopsy. These patients did not have a statistically significant difference in operative time, blood loss or length of hospital stay. In another article, Schwartz *et al.*,[10] described three patients who underwent laparoscopic adrenalectomy in conjunction with simultaneous renal surgery and reported acceptable operative time and hospital stay with no increase in complications.

Bilateral laparoscopic adrenalectomy is now the standard of treatment for patients with Cushing's syndrome.[9] Simultaneous laparoscopic removal of both adrenals has hastened the recovery and reduced wound-related complications, thereby making laparoscopy preferable to the open approach in this subset of patients.[12] Shinbo *et al.*,[13] reported bilateral simultaneous adrenalectomy in a case of pituitary independent macronodular adrenal hyperplasia with less morbidity and early recovery from surgery. In a large series of 39 patients, Thompson *et al.*,[14] found that laparoscopic bilateral adrenalectomy was a safe and effective treatment option for Cushing's disease, with a majority of the patients experiencing considerable improvement in their symptoms and quality of life. Eleven patients in our series underwent bilateral simultaneous laparoscopic adrenalectomy with no significant increase in morbidity as compared to those who underwent unilateral adrenalectomy.

The simultaneous laparoscopic approach for pre-transplant nephrectomy has drastically reduced the waiting period for renal transplantation in patients with end-stage renal disease and uncontrolled hypertension. Wagner *et al.*,[15] analysed 15 patients who underwent bilateral nephrectomy for autosomal dominant polycystic kidney disease with end-stage renal failure and found that the median time from nephrectomy to living donor transplantation was 124 days, which was statistically a significant variable. However, Ismail *et al.*,[16] reported that 63.6% of his patients who underwent simultaneous laparoscopic bilateral native nephrectomy had complications, such as, incisional hernia, urinary fistula, enterocutaneous fistula and so on, which needed an additional surgical procedure. We did not encounter any major complications in this subset of patients.

In our series, the operative duration for combined urological procedures was slightly higher than the unilateral procedures, but this in no way affected the complication rate or morbidity. There were no significant increases in blood loss or hospital stay between these groups. In fact it can safely be assumed that combined procedures have reduced the need for a second anaesthesia, thereby reducing the total operative morbidity.

As we use laparoscopic instruments, the cost of the procedure has to be given much importance from the patient's perspective. In our series, combined procedures had drastically reduced the total expenses when compared to cost of two procedures at two different times.

## CONCLUSION

When compared with a historical cohort of patients undergoing a single laparoscopic procedure, those undergoing simultaneous laparoscopic procedures did not experience a significant increase in perioperative morbidity, duration of hospitalisation or expenses. Provided the basic surgical principles and indication for simultaneous procedures were followed, more patients could enjoy the benefits of minimal access surgery.

Our series is definitely biased in the sense that patients expected to have a high risk of perioperative morbidity during concomitant surgery were operated in multiple sessions. A randomised controlled trial to compare single versus multiple staged surgeries for concomitant pathologic conditions is probably unethical. Hence, the conclusion that could be drawn is that concomitant laparoscopic urologic procedures are feasible in the good risk patient; concomitant surgery reduces the cost and morbidity of another admission and another surgery and reduces the associated anxiety of the patients. Two simultaneous laparoscopic surgeries in a single setting are better than two laparoscopic surgeries done separately.
